# Hyponatremia Presents With Seizure in a Patient With Arnold-Chiari Malformation: A Case Report

**DOI:** 10.7759/cureus.85819

**Published:** 2025-06-11

**Authors:** Mohamed Zuhail K Peediyakkal, Nabil Mahmood, Ashib Thurakkal, Nevin Kannappilly, Sunil Hassan Koya, Saifil Sidhique, Bara M Al-Qudah, Humam E Rajha, Abdulqadir J Nashwan

**Affiliations:** 1 Critical Care Medicine, Hamad Medical Corporation, Doha, QAT; 2 Clinical Imaging, Hamad Medical Corporation, Doha, QAT; 3 Medicine, Hamad Medical Corporation, Doha, QAT; 4 Internal Medicine, Hamad Medical Corporation, Doha, QAT; 5 Medicine, Qatar University, Doha, QAT; 6 Nursing and Midwifery Research, Hamad Medical Corporation, Doha, QAT

**Keywords:** arnold-chiari malformation, hydrocephalus, hyponatremia, seizures, siadh

## Abstract

Arnold-Chiari malformation (ACM) is a neuroanatomical disorder characterized by the displacement of cerebellar tonsils through the foramen magnum. While traditionally associated with a spectrum of neurological symptoms, hyponatremic seizures remain a rare and underrecognized presentation of ACM. This manuscript explores the potential pathophysiological mechanisms linking ACM to hyponatremia and presents a clinical case illustrating this unusual association. Although seizures are a well-documented manifestation of various neurological conditions, their occurrence secondary to hyponatremia in ACM patients has not been extensively reported in the literature. By examining this rare complication, we aim to enhance clinical awareness and deepen the understanding of the underlying mechanisms that may contribute to this phenomenon.

## Introduction

Arnold-Chiari malformation (ACM), commonly called Chiari malformation, is a congenital structural defect characterized by the herniation of cerebellar tissue through the foramen magnum into the spinal canal. This displacement can exert pressure on the brainstem and spinal cord, leading to a broad spectrum of neurological manifestations [1]. ACM is classified into four subtypes (types I-IV) based on anatomical severity and the extent of hindbrain involvement [1]. Among these, Chiari malformation type 1 (CM-1) is the most prevalent and typically presents in late childhood or adulthood with symptoms such as occipital headaches, neck pain, cerebellar dysfunction, myelopathy, and brainstem compression [2,3].

Obstructive hydrocephalus is a notable complication associated with CM-1, resulting from impaired cerebrospinal fluid (CSF) circulation due to structural obstruction at the craniocervical junction. Studies suggest that approximately 10% of CM-1 patients develop hydrocephalus, underscoring a significant pathophysiological link between these conditions [4]. Hydrocephalus, characterized by abnormal CSF accumulation within the cerebral ventricles, elevates intracranial pressure and may lead to severe neurological sequelae if untreated.

Interestingly, hyponatremia has been reported in rare instances of hydrocephalus, likely due to the syndrome of inappropriate antidiuretic hormone secretion (SIADH) or cerebral salt wasting (CSW) secondary to intracranial pathology [5-7]. However, the association between hyponatremic seizures and CM-1-related hydrocephalus remains poorly documented in the literature.

We present a case of an adult patient with previously undiagnosed CM-1 who presented with severe hyponatremia and seizures, later attributed to obstructive hydrocephalus. This report highlights the diagnostic challenges, pathophysiological mechanisms, and management strategies for this rare but critical presentation, emphasizing the importance of early recognition to prevent irreversible neurological damage.

## Case presentation

**Figure 1:**
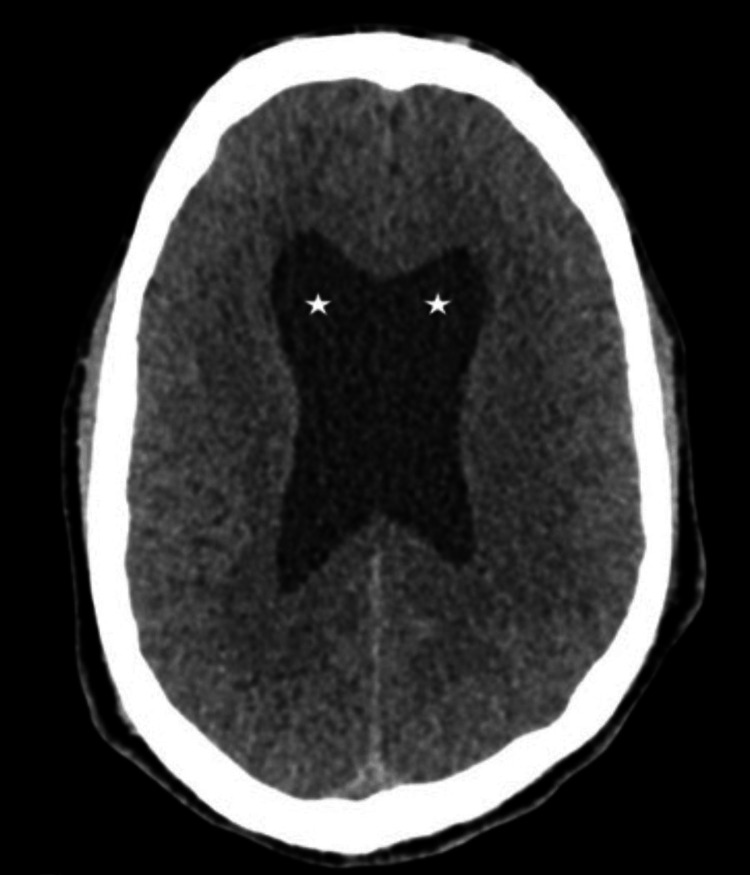
Brain CT showing dilated ventricular system with effacement of extra-axial cerebrospinal fluid (sulcal) spaces (white asterisks)

A 39-year-old gentleman with a known history of hypertension presented to the emergency department with complaints of progressive muscle cramps and headache. Shortly after arrival, he developed generalized tonic-clonic seizures, which were treated with benzodiazepines and levetiracetam. He required endotracheal intubation for airway protection given a low conscious level. Initial computed tomography (CT) of the brain revealed dilated ventricles with effacement of the extra-axial spaces, consistent with obstructive hydrocephalus (Figure 1).

Laboratory investigations at admission revealed hyponatremia (serum sodium 112 mEq/L), along with hypomagnesemia and hypophosphatemia. Further workup for hyponatremia demonstrated low serum osmolality (250 mmol/kg), elevated urine osmolality (417 mmol/kg), and high urine sodium (54 mmol/L), findings consistent with the syndrome of SIADH. Details of laboratory investigations are reported in Table 1.

**Table 1:** Results of blood and urine laboratory investigations on admission β-hydroxybutyrate - beta-hydroxybutyrate; g/dL - grams per deciliter; mmol/kg - millimoles per kilogram; mmol/L - millimoles per liter; ng/mL - nanograms per milliliter; NT pro-BNP - N-terminal pro B-type natriuretic peptide; pg/mL - picograms per milliliter; RBC - red blood cells; U/L - units per liter; WBC - white blood cells; μmol/L - micromoles per liter

Investigation (Unit)	Value	Normal Range
WBC (×10^3^/uL)	26.9	4.0-10.0
RBC (×10^6^/uL)	4.7	4.5-5.5
Hemoglobin (g/dL)	13.4	13.0-17.0
Urinary creatinine (µmol/L)	2,825	124-230
Urinary osmolality (mmol/kg)	417	150-1,150
Urinary sodium (mmol/L)	54	20-40
Urea (mmol/L)	6.8	2.5-7.8
Creatinine (umol/L)	74	62-106
Sodium (mmol/L)	112	133-146
Potassium (mmol/L)	3.9	3.5-5.3
Chloride (mmol/L)	81	95-108
Bicarbonate (mmol/L)	18	22-29
Phosphorus (mmol/L)	1.34	0.80-1.50
Magnesium (mmol/L)	0.69	0.70-1.00
Bilirubin total (µmol/L)	9	0-21
Total protein (g/L)	66	60-80
Albumin level (g/L)	35	35-50
Alkaline phosphatase (U/L)	90	40-129
Alanine aminotransferase (ALT) (U/L)	48	0-41
Aspartate transferase (AST) (U/L)	73	0-40
NT pro-BNP (pg/mL)	197	<125
Creatine kinase (CK) (U/L)	7,062	39-308
β-hydroxybutyrate (mmol/L)	<0.10	0.03-0.30
Myoglobin (ng/mL)	2,982	28-72
C-reactive protein (CRP) (mg/L)	<0.6	0.0-5.0
Osmolality (mmol/kg)	250	275-295
Calcium (mmol/L)	2.42	2.20-2.60
Blood glucose (mmol/L)	6.2	3.3-5.5

Given the severity of hyponatremia and associated seizures, hypertonic saline (3%) was initiated with close monitoring of serum sodium levels to prevent rapid overcorrection.

**Figure 2:**
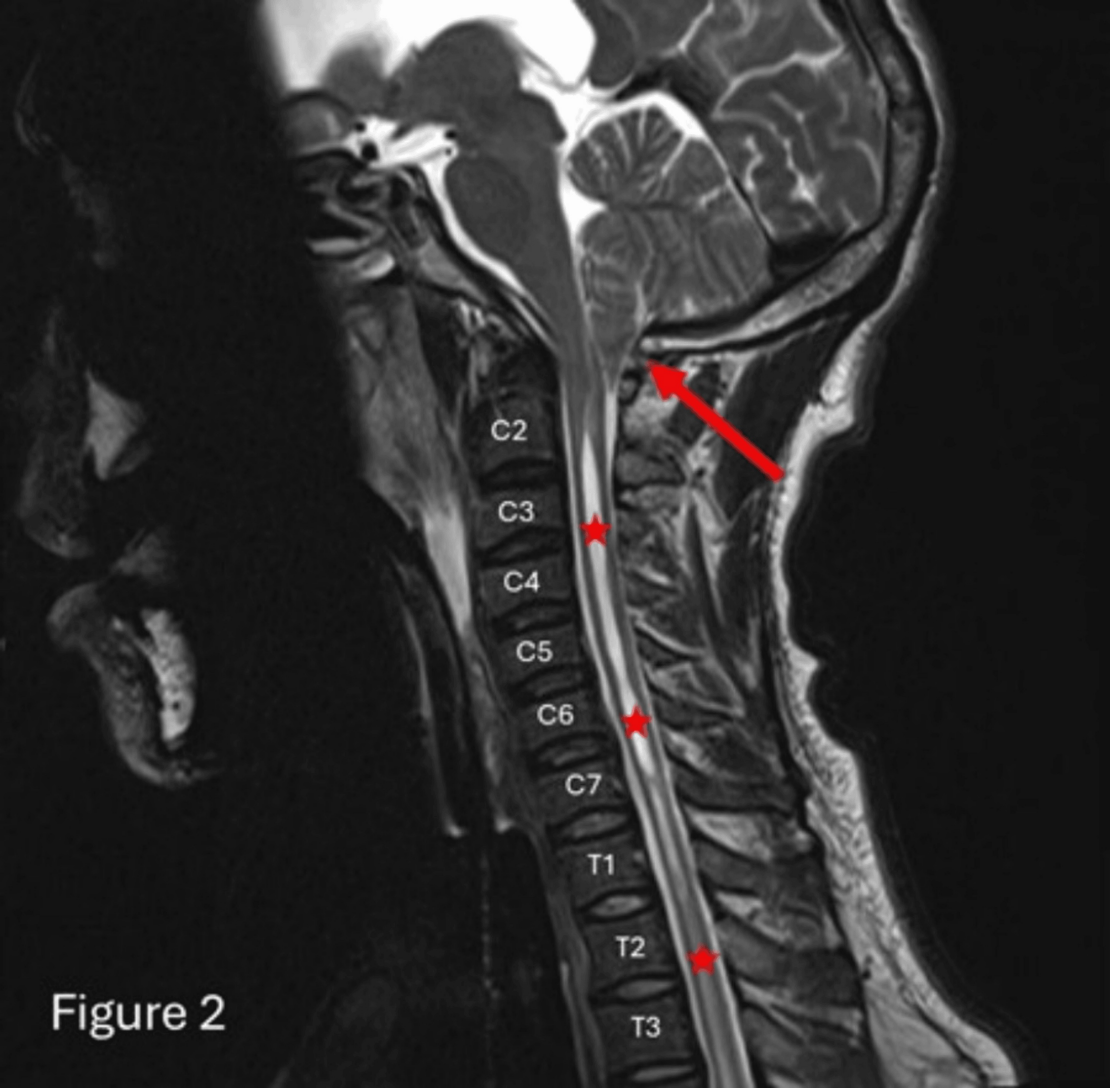
Sagittal T2-weighted MRI Sagittal T2-weighted MRI study of the cervical spine demonstrates a peg-like tonsil (red arrow) herniating across the foramen magnum with associated syrinx of the cervical and thoracic spinal cord (red asterisks) in keeping with Arnold-Chiari I malformation.

Due to the presence of hydrocephalus and seizures, neurosurgical consultation was obtained, and an external ventricular drain (EVD) was emergently placed to relieve intracranial pressure. CSF analysis showed no evidence of infection, including tuberculosis, and toxicology screening was negative for common illicit substances. Subsequent brain magnetic resonance imaging (MRI) confirmed bilateral cerebellar tonsillar herniation extending 12 mm below the foramen magnum, along with an associated cervical syrinx, confirming the diagnosis of CM-1 (Figure 2).

**Figure 3:**
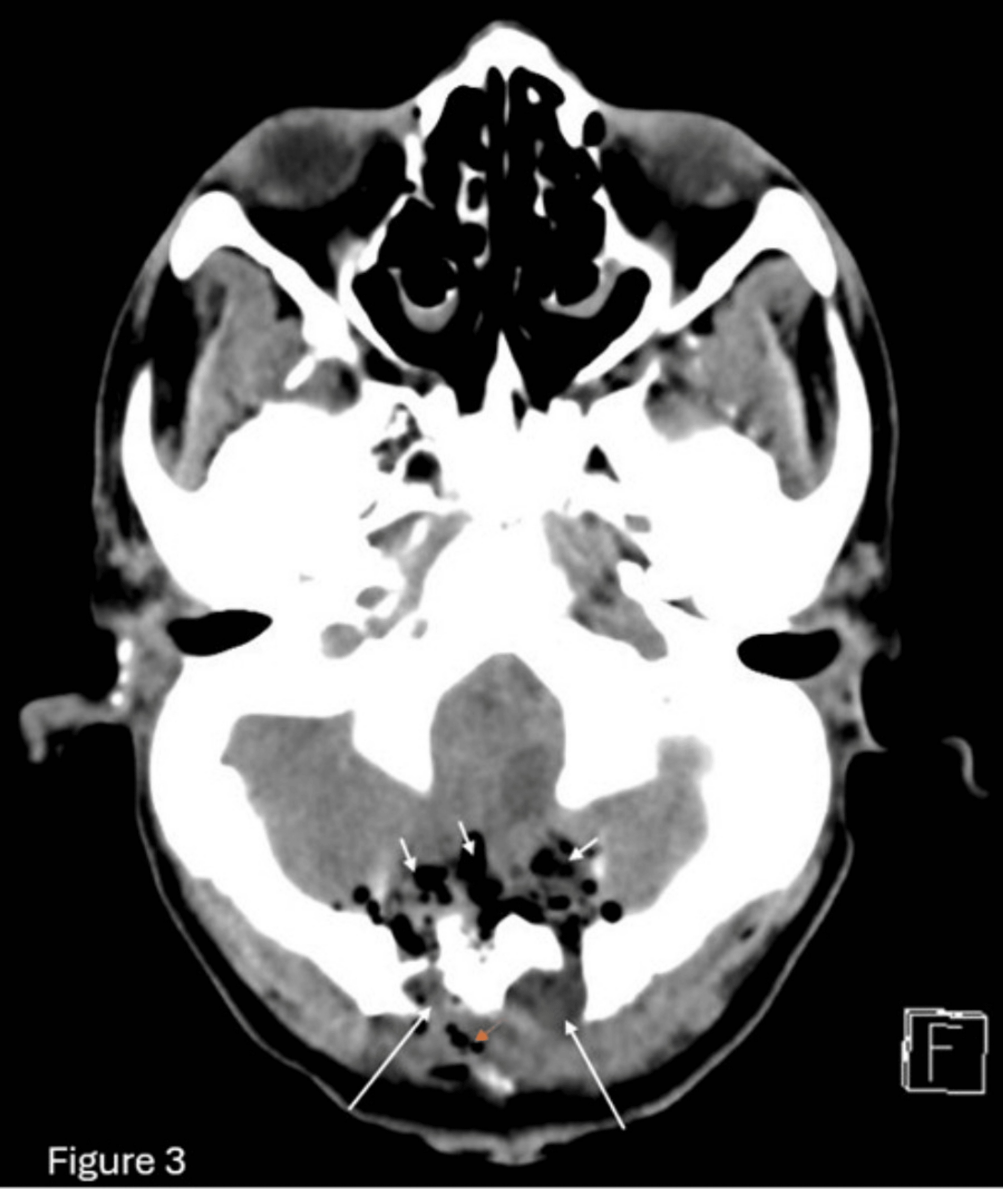
CT image showing postoperative posterior fossa craniotomy with pneumocephalus, surgical emphysema, and mild extracranial soft tissue swelling

Following EVD placement, the patient’s sodium levels gradually normalized, and he remained on mechanical ventilation with minimal sedation. No further seizure episodes were observed after the initial event. A whole-spine MRI was performed to evaluate the extent of syringomyelia, revealing an extensive cervicothoracic syrinx and the previously noted tonsillar herniation (Figure 2). The CT image demonstrates postoperative changes following a posterior fossa craniotomy (Figure 3). Notable findings include the presence of pneumocephalus, characterized by air within the cranial cavity, and surgical emphysema, evidenced by air tracking through the soft tissues. Additionally, there is mild extracranial soft tissue swelling, consistent with expected post-surgical alterations. These findings are typical in the early postoperative period and should be correlated with clinical symptoms and follow-up imaging if indicated.

One week after intubation, the patient was successfully extubated, achieving a Glasgow Coma Scale (GCS) of 11 (E4M6V1) immediately post-extubation. Within 24 hours, he was fully conscious and oriented, though neurological examination revealed 4/5 power in the upper limbs and 2/5 power in the lower limbs, suggesting myelopathic involvement. Given the symptomatic nature of his CM-1, the patient underwent suboccipital decompressive craniectomy with partial C1 laminectomy and duroplasty. The intraoperative and postoperative courses were uneventful, with a follow-up postoperative CT brain showing only minimal pneumocephalus and no evidence of intracranial hemorrhage. He was transferred to the rehabilitation unit four days after surgery, where he exhibited near-normal muscle power on the right side and persistent mild weakness (2/5) in the left foot. After one month of intensive physical therapy, he demonstrated significant functional improvement and was discharged home with outpatient follow-up.

## Discussion

Hyponatremia, defined as a serum sodium concentration below 135 mEq/L, is a common electrolyte disturbance associated with diverse neurological manifestations, including seizures [7]. In patients with pre-existing neurological conditions such as hydrocephalus, the risk of complications like seizures is significantly amplified due to altered intracranial dynamics. Hydrocephalus, characterized by CSF accumulation within the brain's ventricles, elevates intracranial pressure and may exacerbate electrolyte imbalances [8]. In adults with hydrocephalus, hyponatremia may arise from multiple etiologies, including postoperative complications, medication side effects, or endocrine disturbances such as the syndrome of SIADH [9]. The coexistence of hyponatremia and hydrocephalus presents a complex clinical challenge, necessitating meticulous management of both electrolyte abnormalities and intracranial pressure.

In the present case, the patient developed hyponatremia-induced seizures in the setting of hydrocephalus secondary to CM-1. While both hyponatremia and hydrocephalus independently predispose to seizures via disruption of cerebral homeostasis, their convergence in the context of a structural brain anomaly like CM-1 substantially increases diagnostic and therapeutic complexity.

In CM-1, the downward displacement of cerebellar tonsils through the foramen magnum impedes CSF flow, leading to obstructive hydrocephalus [10]. This obstruction elevates intracranial pressure, which not only exerts mechanical stress on neural structures but also disrupts fluid and electrolyte homeostasis. Neurological conditions, particularly those associated with elevated intracranial pressure, predispose patients to SIADH, a leading cause of hyponatremia due to excessive water retention and subsequent dilution of serum sodium [11]. In this patient, chronic CSF flow obstruction from CM-1 likely contributed to both hydrocephalus and dysregulated antidiuretic hormone (ADH) secretion, culminating in profound hyponatremia.

Hyponatremia induces cerebral edema via osmotic imbalance between intracellular and extracellular compartments, increasing neuronal excitability and seizure susceptibility [12-13]. Concurrent hydrocephalus exacerbates this risk by further elevating intracranial pressure and compressing brain structures [14]. The synergistic effect of these conditions in our patient precipitated generalized tonic-clonic seizures, highlighting the critical need for prompt intervention.

Management of hyponatremic seizures in CM-1 with hydrocephalus demands a multidisciplinary approach addressing both electrolyte correction and intracranial pressure regulation. Rapid sodium correction risks osmotic demyelination syndrome; thus, controlled administration of hypertonic saline with frequent serum sodium monitoring is essential [15]. In SIADH, fluid restriction and vasopressin antagonists (e.g., tolvaptan) may aid in restoring sodium balance. While antiepileptic drugs (AEDs) are necessary for seizure management, certain agents (e.g., carbamazepine, oxcarbazepine) may exacerbate hyponatremia. Thus, AED selection should prioritize agents with minimal metabolic interference. Definitive treatment of CM-1-related hydrocephalus often requires surgical decompression. Our patient underwent suboccipital craniectomy with C1 laminectomy and duroplasty, which alleviates cerebellar compression and restores CSF flow [16,17]. Early surgical intervention is critical to mitigate long-term neurological sequelae.

The prognosis in such cases hinges on the timely correction of hyponatremia and the relief of intracranial hypertension. In this patient, rapid sodium normalization and surgical decompression prevented further neurological deterioration. Long-term follow-up is imperative to monitor for recurrent hyponatremia, hydrocephalus progression, or delayed complications of CM-1.

## Conclusions

This case report presents a rare instance of an adult patient with hydrocephalus secondary to ACM, who developed hyponatremic seizures. The report highlights the clinical presentation, diagnostic approach, and therapeutic interventions, underscoring the importance of early recognition and careful management in this complex patient population. It is crucial to recognize that seizures in ACM may result from multifactorial etiologies, including but not limited to hyponatremia, hydrocephalus, and direct cerebellar dysfunction. Maintaining a high index of suspicion and considering Chiari malformation in patients with atypical neurological symptoms and electrolyte disturbances is essential for timely diagnosis and management.
